# The Quorum Sensing Regulated sRNA Lrs1 Is Involved in the Adaptation to Low Iron in *Pseudomonas aeruginosa*

**DOI:** 10.1111/1758-2229.70090

**Published:** 2025-03-27

**Authors:** Dimitra Panagiotopoulou, Natalia Romo Catalán, Max Wilcox, Nigel Halliday, Paolo Pantalone, James Lazenby, Miguel Cámara, Stephan Heeb

**Affiliations:** ^1^ National Biofilms Innovation Centre, School of Life Sciences, Biodiscovery Institute University of Nottingham Nottingham UK; ^2^ National Biofilms Innovation Centre, School of Biological Sciences University of Southampton Southampton UK; ^3^ Revvity Reading UK; ^4^ Research & Development Unilever Port Sunlight UK; ^5^ Science Operations Quadram Institute Bioscience, Norwich Research Park Norwich UK

**Keywords:** iron metabolism, PQS, *Pseudomonas aeruginosa*, pyochelin, quorum sensing, sRNA

## Abstract

Iron is an essential nutrient for microbial growth. The opportunistic pathogen *Pseudomonas aeruginosa* can survive under diverse conditions, including iron‐depleted environments with the aid of small non‐coding RNAs (sRNAs). *P. aeruginosa* also uses three quorum sensing (QS) systems: Las, Rhl and Pqs, to coordinate virulence and infection establishment at the population level. The aim of this study is to investigate the role of the sRNA Lrs1, the gene of which is positioned within the promoter of the Pqs biosynthetic operon *pqsABCDE.* Transcriptomics and phenotypic assays indicate that Lrs1 downregulates the production of the siderophore pyochelin but not pyoverdine, and that *lrs1* regulation itself is dependent on iron availability. Although Lrs1 has been implicated in a positive feedback loop with the transcriptional regulator LasR in the strain PA14, the present findings indicate that this is not the case in PAO1‐L in the tested conditions. Transcription of Lrs1 is dependent on quorum sensing, predominantly on RhlR with an auxiliary effect by PqsE. Furthermore, the Pqs system and phenazine production are modulated by Lrs1 only under iron limitation. This study identifies Lrs1 as a new QS‐dependent post‐transcriptional regulator in low iron, highlighting its importance in environmental adaptation in *P. aeruginosa* .

## Graphical abstract

The quorum sensing regulated sRNA Lrs1 participates in the adaptation of *Pseudomonas aeruginosa* in low iron by downregulating the siderophore pyochelin, the Quorum Sensing system PQS, and its associated virulence factor phenazines.

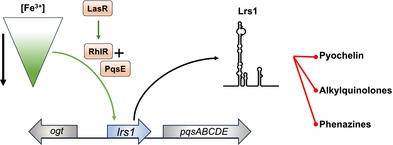

## 1 Introduction

*Pseudomonas aeruginosa* is an opportunistic pathogen with the ability to colonise diverse environments. It is one of the most common healthcare‐associated pathogens, causing antibiotic‐resistant infections and a major morbidity risk factor for cystic fibrosis (CF) patients (CDC 2019; Malhotra et al. 2019). The pathogenesis of *P. aeruginosa* is controlled at the population level via the coordinated action of three quorum sensing (QS) systems, the Las, Rhl, and Pqs systems. The Las and the Rhl QS systems rely on the production and sensing of *N*‐acyl‐homoserine lactones as their cognate signals. The complex LasR—*N*‐(3‐oxododecanoyl)‐L‐homoserine lactone (3OC_12_‐HSL) activates the expression of genes involved in biofilm formation and virulence, such as that encoding the secreted elastase LasB (Williams and Cámara 2009). Homeostasis in the activation of the Las system is preserved by the negative transcriptional regulator RsaL, which binds to the bidirectional promoter of *rsaL*‐*lasI*, repressing both its own expression and that of the 3OC_12_‐HSL synthetase gene *lasI* (Rampioni et al. 2007). The RhlR transcriptional regulator forms a complex with the *N*‐butanoyl‐L‐homoserine lactone (C_4_‐HSL) controlling the production of virulence factors including hydrogen cyanide, pyocyanin, and determinants of biofilm formation (Williams and Cámara 2009). The third QS system, known as the Pqs system, is driven by 2‐alkyl‐4(1*H*)‐quinolones (AQ) signal molecules. For the biosynthesis of these molecules, chorismate is converted to anthranilate via the action of PhnAB or TrpEG and subsequently to AQs via the products of the *pqsABCDE* operon and *pqsH* (Farrow and Pesci 2007). The PqsR transcriptional regulator binds either 2‐heptyl‐4‐hydroxyquinoline (HHQ) or 2‐heptyl‐3‐hydroxy‐4‐quinolone, also referred to as the *Pseudomonas* quinolone signal (PQS), and enhances the expression of the *pqs* operon, as well as the production of multiple virulence traits including pyocyanin and the release of eDNA contributing to biofilm architecture (Xiao et al. 2006a; Rampioni et al. 2010). However, RhlR negatively regulates both the *pqsABCDE* operon and the *pqsR* gene (Xiao et al. 2006b). Besides its role in QS, the PQS molecule has iron‐chelating properties, restricting access to environmental iron by competing bacteria, further enhancing the adaptation of *P. aeruginosa* infections in a polymicrobial environment (Bredenbruch et al. 2006; Diggle et al. 2007).

The QS systems can also respond to environmental signals at the post‐transcriptional level through the action of small non‐coding RNAs (sRNAs). These sRNAs can act by base pairing with target mRNAs, altering their translational rate or stability, and these interactions may be dependent on the RNA chaperone Hfq (Wagner and Romby 2015). Under iron limitation, the expression of the Pqs system is increased by the indirect action of the PrrF1 and PrrF2 sRNAs (Oglesby et al. 2008). These two redundant sRNAs repress *antR*, encoding the regulator of anthranilate degradation, redirecting anthranilate, the alkyl‐quinolone precursor, towards the biosynthesis of PQS. This post‐transcriptional regulation is antagonised by the Carbon Catabolism Regulator sRNA CrcZ, allowing for more anthranilate to be degraded by products of the AntR‐induced genes *antABC* and *catBCA* towards the TCA cycle (Sonnleitner et al. 2017). CrcZ sequesters Hfq, preventing the Hfq‐mediated riboregulation of *antR* by PrrF1 and PrrF2, resulting in indirect increased translation of *antR* and so expression of *antABC*. The main role of CrcZ is adaptation of *P. aeruginosa* to non‐preferred carbon sources. CrcZ, along with CrcA, occludes the Hfq‐Crc repression of catabolic genes mRNAs in the absence of preferred carbon sources such as succinate (Sonnleitner et al. 2023). The sRNA PhrS positively affects the Pqs QS system from two fronts; it increases the translation rate of *pqsR* while repressing that of *antR*, hence more anthranilate is directed to PQS production (Sonnleitner et al. 2011; Gebhardt et al. 2023).

In infection sites such as the CF lung, pathogens compete for access to iron (Hunter et al. 2013; Konings et al. 2013). *P. aeruginosa* possesses multiple systems for iron uptake from the environment aiding the establishment of acute and chronic infections (Cornelis and Dingemans 2013; Konings et al. 2013). *P. aeruginosa* produces two siderophores, pyoverdine and pyochelin, with high and low affinity for iron, respectively (Brandel et al. 2012; Cornelis and Dingemans 2013). It is hypothesised that the low iron affinity pyochelin is produced first, while pyoverdine, with the more energy‐demanding biosynthetic process, is reserved for iron scarcity (Dumas et al. 2013; Cunrath et al. 2020). Pyochelin biosynthesis, like PQS, starts with chorismic acid. Chorismate is converted to salicylate, followed by condensation with two cysteines (Reimmann et al. 1998). The pyochelin biosynthetic operons *pchDCBA* and *pchEFGHI*, along with the cognate Fe(III)‐pyochelin receptor gene *fptA*, are induced by the transcriptional regulator PchR bound to the ferripyochelin complex (Michel et al. 2005). When iron is low, the sRNAs PrrF1,2 negatively regulate the mRNAs of non‐essential iron‐containing proteins, preserving iron for essential processes (Wilderman et al. 2004). When iron is present, both transcription of PrrF1,2 and production of the siderophores are repressed by the iron regulator Fur, while the iron storage protein BfrB is upregulated, thus maintaining iron homeostasis (Wilderman et al. 2004; Cornelis et al. 2009).

The sRNA Lrs1 transcribed within the *pqsA* promoter region has been shown to be involved in a positive regulatory feedback loop with LasR, in surface‐attached cells of the *P. aeruginosa* strain PA14 (Chuang et al. 2019). Deletion of *lrs1* resulted in reduced transcript levels of *lasR* in both planktonic and surface‐attached cells, with a more pronounced effect in the latter. This deletion also abolished elastase production in planktonic cultures. However, in contrast to these observations, a previous RNA‐seq analysis of the same Δlrs1 deletion mutant in planktonic cells revealed that neither the expression of *lasR* nor *lasB* was altered compared to the wild type (Wurtzel et al. 2012). In this study, we show how we have independently identified *lrs1* in the *P. aeruginosa* PAO1‐L strain through an RNA‐seq analysis and how we sought to address the discrepancies mentioned above by investigating the role that Lrs1 has in the QS systems of this strain. We found that in PAO1‐L, Lrs1 does not significantly impact the Las and Rhl QS systems in the conditions tested. However, the Pqs QS system seems to be conditionally affected, with no observed effect when grown in LB but a downregulation by Lrs1 in iron scarcity. Furthermore, we found that Lrs1 is involved in the regulation of the iron acquisition systems, particularly of the siderophore pyochelin, and that this regulation is dependent on iron availability. This regulation may be due to the participation of Lrs1 in balancing metabolic flow, deprioritising pyochelin, PQS and phenazine production in favour of central metabolism and aromatic amino acid biosynthesis. This is the first demonstration of a regulatory connection between the Pqs QS system and the siderophore pyochelin by a post‐transcriptional regulator, offering novel mechanistic insight into the regulation of iron acquisition in *P. aeruginosa* .

## 2 Results

### 2.1 Lrs1 Appears in Multiple Transcript Forms

**FIGURE 1:**
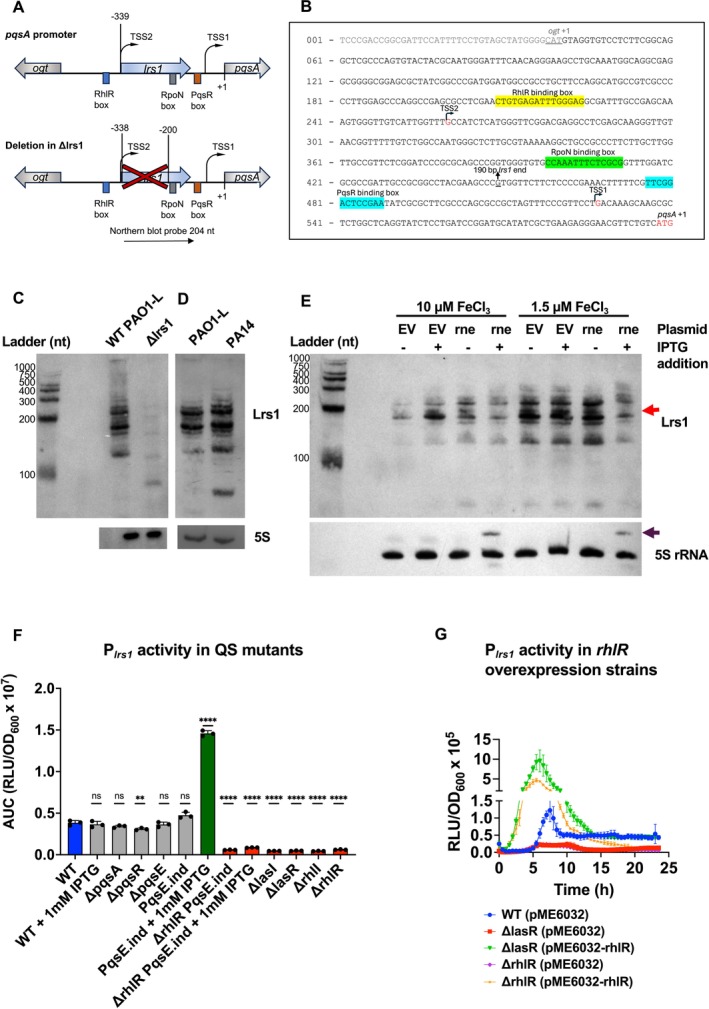
Lrs1 abundance is dependent on RNase E and the *lrs1* promoter activity on the three QS systems. (A) Construction of *lrs1* deletion mutant. Schematic representation of the intergenic region between *pqsA* and the upstream *ogt* gene showing the position of *lrs1* starting at TSS2, 339 bp upstream of the *pqsA* translational start site (+1). The positions of RhlR, PqsR and RpoN binding boxes are also depicted with rectangles. Of the 190 bp of *lrs1*, only the first 138 bp were deleted, leaving the TSS2 unaffected. This was done to preserve the RpoN binding site found in the *pqsA* promoter (Shao et al. 2018). (B) Genetic organisation of the intergenic region between *pqsA* and *ogt*. The TSS1 and TSS2 are indicated with bent arrows. The translational start site of *pqsA* is in red and that of *ogt* is underlined. The 190 bp experimentally verified end of *lrs1* (Wurtzel et al. 2012) is underlined. The RhlR binding box is highlighted in yellow, the RpoN binding box is highlighted in green, and the PqsR binding box in blue. (C) Northern blot of Lrs1 in PAO1‐L after 12 h of incubation in LB. (D) Northern blot of Lrs1 in PAO1‐L and PA14 after 12 h of incubation in LB. (E) RNase E is involved in processing and abundance of Lrs1. Northern blot of Lrs1 from cells grown at 10 and 1.5 μΜ iron. Samples were collected 16 h post‐inoculation in M9 medium with glucose and the iron concentration indicated. EV: empty vector, rne: pBx‐sgRNA‐rne expressing plasmid, constitutively transcribing the guide RNA for the RNase E gene *rne*. IPTG addition induces the expression of the catalytically inactive (dead) Cas9 from the P_*lac*_ inducible promoter. The *rne* gene is knocked down and the levels of RNase E are reduced in the cells transcribing the sgRNA targeting *rne* from pBx‐sgRNA‐rne plasmid. Where indicated, IPTG was added at 1 mM final concentration at the beginning of the culture. 5S rRNA was used as loading control. Red arrow indicates the lack of one Lrs1 band in the IPTG‐induced expression of dCas9 in the pBx‐sgRNA‐rne harbouring strain. Purple arrow represents the presence of a higher molecular weight band of 5S rRNA in induced strains with the pBx‐sgRNA‐rne plasmid, indicative of loss of 5S rRNA processing due to RNase E knockdown. (F) Transcriptional activity of the *lrs1* promoter (P_*lrs1*_) in different QS mutants. Approximately 500 bp upstream of *lrs1* were cloned upstream of the bioluminescence reporter operon *luxCDABE* and promoter activity was measured over 24 h in PAO1‐L WT and QS mutants in LB. Luminescence was measured in Relative Luminescence Units (RLU) and was normalised by growth (OD_600_). The Area Under the Curve (AUC) of each curve has been plotted here. Error bars represent the standard deviation of three biological replicates with three technical replicates. One‐way ANOVA was used as statistical test. ns: not significant. *p ≤ 0.05, **p ≤ 0.01, ***p ≤ 0.001, ****p ≤ 0.0001. (G) Transcriptional activity of P_*lrs1*_ in strains overexpressing *rhlR*. Error bars represent three biological replicates with two technical replicates.

Lrs1 is an sRNA that lies upstream of the *pqsA* coding sequence. BlastN analysis of Lrs1 showed its existence only within *P. aeruginosa* , with the shortest size of a homologue being 190 nt long in strain PA14 (Wurtzel et al. 2012). In our lab, Lrs1 was independently identified in an RNA‐seq analysis carried out in strain PAO1‐L after 8 and 12 h of incubation in LB (Figure S1A). In the *pqsA* promoter (P_*pqsA*_) region, two transcriptional start sites (TSS) have been identified, TSS1 and TSS2 (Dötsch et al. 2012) (Figure 1A,B). Transcription from TSS2 appeared to continue beyond the 190 nt end point of *lrs1*, suggesting that Lrs1 may exist as a longer transcript (Figure S1B). To investigate this possibility, Northern blot was conducted in the PAO1‐L wild type (WT) strain after 12 h of incubation in LB to match the time point of the RNA‐seq and compared to the bands produced by an *lrs1* deletion mutant (Figure 1C). Indeed, more than one band was observed in the PAO1‐L compared to the *lrs1* mutant, suggesting the presence of transcripts of different sizes driven by the TSS2 promoter. The bands observed in Δlrs1 did not present the same pattern as in the WT. Since *lrs1* was only partially deleted and the Northern blot probe binds to a further 52 nt, these bands are products transcribed from TSS2, as deletion of the entire *lrs1* completely eliminated these bands (Figure S1D). The presence of these bands indicates that transcription from TSS2 had not been affected by the deletion of *lrs1*. Detection of Lrs1 in PA14 also revealed the same bands as in the PAO1‐L WT, suggesting that Lrs1 is present in multiple forms in *P. aeruginosa* , and that this is not strain‐specific (Figure 1D).

### 2.2 Abundance of Lrs1 Is Dependent on RNase E

Often, sRNAs are transcribed as longer precursor transcripts before they are processed post‐transcriptionally by RNase E to adopt their shorter, mature forms. RNase E preferentially cuts RNAs in single‐stranded regions that are enriched with poly‐U, and in *Salmonella typhimurium* , RNase E recognises the pattern RNWUU where R is G/A, N any nucleotide, and W is A/U (Chao et al. 2017). Looking at the predicted secondary structures of Lrs1 and the long 5′ UTR starting from TSS2, three single‐stranded regions contain motifs resembling recognition sites for RNase E (Figure S2). Therefore, it was hypothesised that RNase E may be involved in the processing of Lrs1.

To explore this hypothesis, RNase E was depleted from the cells using a CRISPR interference approach (CRISPRi) previously developed for *P. aeruginosa* (Tan et al. 2018) and Northern blots were performed to evaluate its effect on Lrs1. The experiment was conducted in both high and low iron availability, as a connection between Lrs1 and iron is shown later in this study. The abundance of Lrs1 was significantly reduced in RNase E‐depleted cultures in low iron conditions, and less so in high iron (Figure 1E). Although reduced RNase E activity often results in increased abundance of RNAs in some cases, it may also have the opposite effect (Stead et al. 2011; Mackie 2013). Additionally, at least one of the bands of Lrs1 disappears in the *rne‐*repressed cells compared to cells carrying the empty vector, implicating RNase E in the post‐transcriptional processing of Lrs1. In conclusion, RNase E is essential for normal levels and post‐transcriptional processing of Lrs1.

### 2.3 Lrs1 Is Regulated by the Three QS Systems of *P. aeruginosa* PAO1‐L

The gene *lrs1* overlaps with the *pqsA* promoter and its transcription is initiated from the distal transcriptional start site of *pqsA*, the TSS2 (Figure 1A,B). However, little is known about the effect of the three QS systems on the regulation of transcription from TSS2, besides the positive regulation by LasR and RhlR (Wurtzel et al. 2012; Brouwer et al. 2014). To this end, the 512 bp upstream of TSS2, including the RhlR binding box and TSS2, named here as the *lrs1* promoter (P_*lrs1*_), was transcriptionally fused to the *luxCDABE* operon. The activity levels of the promoter were monitored throughout growth in different QS mutants and were compared to the WT.

The activity of the *lrs1* promoter was impacted by deletions in the Las and Rhl QS systems but not the Pqs QS system (Figures 1F and S3). Deletion of *lasR, lasI, rhlR* or *rhlI* silenced the promoter (Figures 1F and S3C,D). Considering the positive regulation of *rhlR* by LasR (Latifi et al. 1996), it was hypothesised that the effect of *lasR* deletion may be indirect, through diminished levels of RhlR. To investigate this, both *lasR* and *rhlR* mutants were complemented with a vector overexpressing *rhlR*. Indeed, overexpression of *rhlR* significantly increased the activity of P_*lrs1*_, including in the *lasR* mutant, indicating that RhlR is the primary regulator of the promoter and the LasR effect is indirect (Figure 1G). It has been suggested that the effector protein of the Pqs QS system, PqsE, and RhlR physically interact, and that interaction alters the DNA binding affinity of RhlR, thus affecting its regulatory activity (Borgert et al. 2022; Feathers et al. 2022). For this reason, the effect of *pqsE* on P_*lrs1*_ was also tested. Deletion of *pqsE* did not impact the promoter activity (Figures 1F and S3A). In contrast, overexpression of *pqsE* from the IPTG‐inducible P_*tac*_ promoter significantly increased the activity levels of P_*lrs1*_, an effect lost upon deletion of *rhlR* in this mutant (Figures 1F and S3B). In conclusion, the *lrs1* promoter is indirectly regulated by the Las QS system, primarily regulated by the Rhl QS systems, with PqsE having a positive yet auxiliary impact on this promoter in PAO1‐L in LB.

### 2.4 Lrs1 Does Not Participate in the Regulation of the QS Systems

Considering the location of *lrs1*, it is plausible that a regulatory link between Lrs1 and the *pqs* operon may exist. The activity of a *pqsA‐luxCDABE* reporter was not affected at the transcriptional but only slightly at the translational level when *lrs1* was overexpressed (Figure S4A–D). Quantification of PQS, HHQ, HQNO, and their precursor anthranilate in culture supernatants overexpressing *lrs1* showed no significant difference when compared to the strain with the empty vector (Figure S4F–I). It was noted that deletion of *lrs1* reduced the production of HHQ while increasing the levels of its precursor anthranilate, but this phenotype could not be complemented by the overexpression of *lrs1* from the pKH6 plasmid. Both the WT and Δ*lrs1* were whole genome sequenced with no single nucleotide polymorphisms (SNPs) detected between the two strains, suggesting that the HHQ reduction was attributed solely to the *lrs1* deletion. Additionally, the deletion of *lrs1* impacted the transcriptional output of P_*pqsA*_ (Figure S4J,K), indicating that reduced HHQ production may be due to reduced activity from the *pqsA* promoter and not an effect attributed to Lrs1. Therefore, Lrs1 does not seem to have a major impact on the regulation of the *pqs* operon under the tested conditions.

Previous work on Lrs1 found a positive regulatory link between Lrs1 and the Las QS system in PA14 (Wurtzel et al. 2012; Chuang et al. 2019). Therefore, the connection of Lrs1 to the Las and Rhl QS systems was investigated in the strain PAO1‐L. Lrs1 overexpression did not affect the transcriptional activity of the *lasR, lasI* and *rhlI* promoters, nor did it impact the levels of their respective QS signal molecules (Figure S5). Furthermore, the LasR‐regulated virulence factor elastase (LasB) was not affected by Lrs1 (Figure S5D). Therefore, Lrs1 does not seem to be involved in the regulation of Las or Rhl QS systems in the strain PAO1‐L under the tested conditions.

### 2.5 Lrs1 Is Involved in the Regulation of Iron Acquisition Systems in PAO1‐L

To unravel the role of this sRNA in PAO1‐L, transcriptomic analysis was performed in the strain overexpressing *lrs1* in *trans* and compared to that of the WT carrying the empty vector. The *lrs1* deletion mutant was not included here to avoid any confounding effects from reduced transcription of the *pqsABCDE* operon. The two strains were cultured in LB in the presence of L‐arabinose for the induction of *lrs1*, and samples were collected from both exponential and stationary phases for analysis (Figure S6A).

**FIGURE 2:**
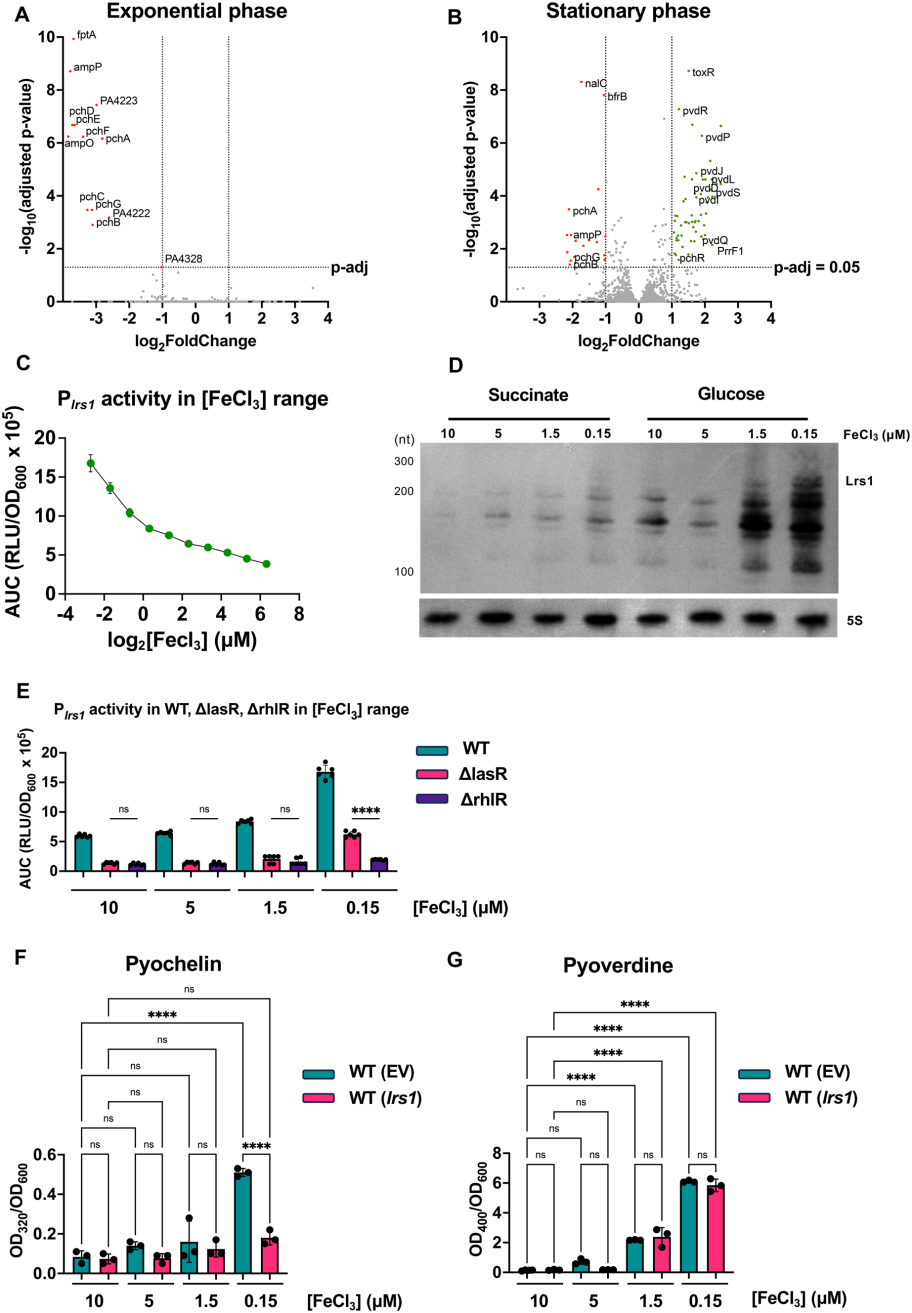
Lrs1 participates in regulation of iron uptake through differential regulation of the siderophores pyochelin and pyoverdine. Volcano plots of the differentially regulated genes in PAO1‐L overexpressing *lrs1* at (A) exponential and (B) stationary phase in LB. Genes with at least −1 ≥ log2FoldChange or log2FoldChange ≥ 1 and *p* adjusted ≤ 0.05 were considered differentially regulated. Negatively affected genes are coloured red and positively affected are green. (C) Promoter activity of P_*lrs1*_ in range of FeCl_3_ concentrations. WT with the P_*lrs1*_ bioluminescent reporter was monitored for 48 h in M9 minimal medium with succinate as carbon source. The promoter activity was measured as Relative Luminescent Units normalised by growth (RLU/OD_600_) and then the area under the curve (AUC) was plotted here. The FeCl_3_ concentrations ranged from 80 to 0.155 μΜ following 2‐fold serial dilution steps. Error bars represent standard deviation of three biological replicates with two technical replicates. (D) Northern blot of Lrs1 in range of iron concentrations. PAO1‐L was grown in M9 minimal medium with glucose or succinate and 10, 5, 1.5 and 0.15 μΜ FeCl_3_ for 16 h when RNA samples were collected. 5S rRNA was used as a loading control. (E) *lrs1* promoter activity (P_*lrs1*_) in ΔlasR or ΔrhlR compared to PAO1‐L WT at 10, 5, 1.5 and 0.15 μΜ FeCl_*3*_. The activity of the P_*lrs1*_ bioluminescent reporter was measured throughout growth for 48 h and the Area Under the Curve (AUC) was plotted here. Error bars represent standard deviation of three biological replicates with two technical replicates. (F) Pyochelin and (G) Pyoverdine quantification in culture supernatants in presence of 0.2% L‐arabinose in M9 minimal medium with glucose. Error bars represent standard deviation of three biological replicates. Two‐tail ANOVA was used as statistical test. ns: not significant. *p ≤ 0.05, **p ≤ 0.01, ***p ≤ 0.001, ****p ≤ 0.0001.

The transcriptomic analysis revealed a strong connection between Lrs1 and iron acquisition genes (Figure 2A,B). Of the 58 upregulated genes in stationary phase, 43 were categorised by gene ontology enrichment analysis in connection to iron acquisition and transport, which was also confirmed by comparing the present dataset to a previous transcriptomic analysis on iron depletion (Ochsner et al. 2002) (Figure S6B,C, Table S1).

Among the differentially regulated genes, eight out of the total 14 extracytoplasmic functioning sigma factors (ECFσ) participating in the iron stringency response were identified, displaying higher transcript levels when *lrs1* was overexpressed. Besides the ECFσ factors, their collective regulome was also affected by Lrs1. More than half of the positively differentially regulated genes (35/58) belong to the regulomes of one of these ECFσ factors (Table S1). Lrs1 may not directly affect all these genes, but its overall impact leads to the activation of the iron acquisition response.

### 2.6 Lrs1 Is Highly Transcribed in Low Iron Conditions and This Expression Is Dependent on RhlR and Indirectly on LasR

To corroborate the importance of Lrs1 in iron stringency, the promoter activity of *lrs1* was measured using a range of iron concentrations in the medium. The activity of P_*lrs1*_ was inversely correlated to the iron concentration, showing a strong regulation of Lrs1 by iron levels (Figure 2C). Northern blot analysis confirmed that Lrs1 transcript levels were higher in lower iron concentrations (Figure 2D). The impact of iron depletion on *lrs1* transcription was also dependent on the carbon source used in minimal medium, since Lrs1 was more abundant when the less preferred carbon source glucose was used in place of succinate (Figure 2D).

The activity of the *lrs1* promoter was also studied in the absence of *rhlR* or *lasR*, considering their regulatory connection (Figure 1F). The promoter activity was dependent upon the presence of both regulators in low iron conditions, since deletion of these genes noticeably reduced *lrs1* transcription, although the *lasR* deletion in the lowest iron concentration was less impactful than the *rhlR* deletion (Figure 2E). Additionally, the dependency of the *lrs1* promoter on the iron regulator Fur was explored and compared to the Fur‐regulated promoter of PrrF1 sRNA (P_*PrrF1*_) (Wilderman et al. 2004). Unlike P_*PrrF1*_, the activity levels of P_*lrs1*_ were not affected by the addition of the iron chelator 2,2′‐Dipyridyl (DIP) in the medium, indicating that this promoter is not under the regulation of Fur (Figure S6F). Taken together, unlike the iron‐responsive PrrF1,2 sRNAs, Lrs1 remains QS‐regulated in low iron conditions.

### 2.7 Lrs1 Decouples the Production of the Siderophores Pyochelin and Pyoverdine

In both exponential and stationary phases, *lrs1* induction downregulated the pyochelin biosynthetic genes *pchDCBA* and *pchEFGHI*, one of the two siderophores produced by *P. aeruginosa* (Figure 2A,B). However, the biosynthetic gene clusters for the second siderophore, pyoverdine, were upregulated in the stationary phase when *lrs1* was overexpressed. In previous studies, the biosynthesis of the two siderophores followed the same trend, being both upregulated or downregulated in response to iron‐repleted or depleted conditions (Ochsner et al. 2002; Palma et al. 2003). Here, Lrs1 seems to be involved in the decoupling of the expression of the two siderophores.

To investigate the involvement of Lrs1 in the regulation of the two siderophores, the levels of pyochelin and pyoverdine were measured in four different iron concentrations in M9 medium: 10, 5, 1.5 and 0.15 μΜ FeCl_3_. These concentrations were chosen as high, intermediate, low and very low iron conditions. Overexpression of Lrs1 reduced pyochelin levels only at the lowest iron concentrations without affecting the levels of pyoverdine, confirming the differential regulation of the two siderophores by Lrs1, a phenomenon not dependent on the carbon source used (Figures 2F,G and S6H,I). There is a possibility that the increase in iron uptake genes and pyoverdine biosynthesis in the RNA‐seq was in response to reduced pyochelin production during the exponential phase of growth. Maybe, the reduced influx of iron from ferripyochelin was perceived by the cells as a lack of iron, resulting in the expression of iron uptake genes.

### 2.8 Lrs1 May Be Involved in Other Metabolic Pathways

RNA‐seq uncovered a regulatory connection between Lrs1 and iron acquisition. Fur would have been a probable target of Lrs1; however, only a specific subset of the Fur regulome was affected by Lrs1, and the siderophore dysregulation did not agree with this hypothesis (Cornelis et al. 2009). Considering the downregulation of the pyochelin gene clusters in both exponential and stationary phases, it was thought that Lrs1 may impact the expression of the pyochelin transcriptional inducer PchR (Michel et al. 2005). However, *pchR* transcription was unaffected in the exponential phase and was upregulated in the stationary phase (Figure 2A,B and Table S1), it appears less likely that Lrs1 is involved in the regulation of *pchR*.

To explore which RNAs may be directly targeted by Lrs1, GRIL‐seq (Global sRNA Target Identification by Ligation and Sequencing) was employed (Han et al. 2016). Using GRIL‐seq, RNAs in direct contact or in proximity to the sRNA of interest can be identified, pointing towards direct RNA targets. The RNAs in contact with the sRNA are ligated to the sRNA by an heterologously expressed T4 RNA ligase, creating chimeric RNAs. In this study, samples of two biological replicates from both exponential and stationary phases were collected and searched for Lrs1 chimeras. The chimeras identified in both biological replicates were considered more likely targets of Lrs1.

None of the pyochelin genes were identified as direct targets of Lrs1 (Tables S2–S4). On the contrary, the products of the identified mRNAs participate in metabolic pathways including virulence factor production and regulation, carbon metabolism, and adaptation to low oxygen conditions, among others. One of the identified potential targets was the *rsaL* mRNA. Although no impact on the *las* QS system by Lrs1 was observed in PAO1‐L (Figure S5), *rsaL* may have been the regulatory link between *lrs1* and *lasR* in PA14 (Wurtzel et al. 2012; Chuang et al. 2019).

In addition to the mRNAs, Lrs1 was also found to bind to three sRNAs, CrcZ, PhrS and ErsA. It has been previously shown that at least PhrS interacts with both CrcZ and ErsA, and that CrcZ interacts with PrrF1 in vivo (Han et al. 2016; Zhang et al. 2017; Gebhardt et al. 2023). These RNAs potentially interact directly with Lrs1 and are not necessarily impacted in LB. It is likely that Lrs1 indirectly affects pyochelin biosynthesis through these targets.

Comparison of the potential targets identified with GRIL‐seq to the differentially regulated genes from the RNA‐seq resulted in *bfrB* being the only commonly identified gene (Figure S7A,B). BfrB is the major bacterioferritin of *P. aeruginosa* and is responsible for iron storage (Eshelman et al. 2017; Rivera 2017). Lrs1 interacted with the 5′ UTR of *bfrB*, near its predicted RBS. Although the two RNAs interacted in vitro, translational repression of *bfrB* by Lrs1 could not be verified (Figure S7C–E). It is possible that either Lrs1 does not majorly impact *bfrB*, or other regulators are masking its effect.

sRNA‐mRNA interactions initiate at a short regions of perfect complementarity between the sRNA and the mRNA termed seed region (Wagner and Romby 2015). Mapping of the seed regions on the mRNA that interacted with Lrs1 revealed that Lrs1 does not only interact with the 5′ UTR of its targets, but also with other regions and with a preference for the 3′ UTR (Figure S8). Lrs1 may not only interact with a diverse number of mRNAs, but the mechanism of action may vary for each mRNA target. There are only a few instances where sRNAs also interact with other regions of their targets besides the 5′ UTR, as was the case of PrrF1 and sKatA (Han et al. 2016).

### 2.9 Lrs1 Overexpression Favours Amino Acid Biosynthesis Over Pyochelin and Alkylquinolones in Glucose M9 Medium

**FIGURE 3:**
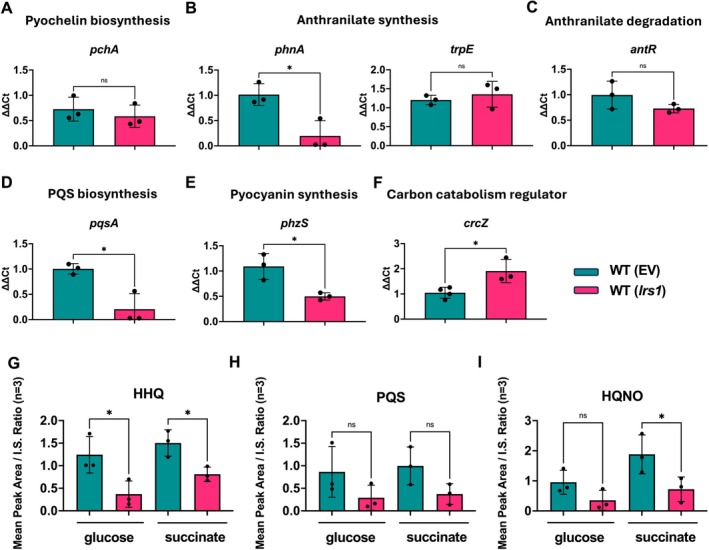
Lrs1 may participate in metabolic rewiring in low iron conditions. RT‐qPCR analysis of genes participating in (A) pyochelin biosynthesis, (B) Anthranilate synthesis, (C) Anthranilate degradation, (D) PQS biosynthesis, (E) pyocyanin production, (F) Carbon catabolism regulation in the WT and the *lrs1* overexpressing strain growing in M9 minimal medium with glucose and in the presence of 0.15 μΜ FeCl_3_. (G) HHQ, (H) PQS, (I) HQNO quantification in 0.15 μΜ FeCl_3_ in presence of either glucose or succinate. Cells were grown for 20 h, at which point samples were collected. Error bars represent standard deviation of three biological replicates. T‐test was used for statistical analysis. ns: not significant. *p ≤ 0.05; **p ≤ 0.01.

To further confirm the involvement of Lrs1 in the regulation of pyochelin production, RT‐qPCRs were conducted between the WT with the empty vector and WT overexpressing *lrs1* in 0.15 μM iron in M9 with glucose. Unexpectedly, there was no change in the mRNA levels of the pyochelin biosynthetic genes between the two strains (Figures 3A and S9D). Probably, derepression of the *pch* genes by Fur in low iron has a more dominant effect on their transcript levels than Lrs1 in these conditions.

Considering that Lrs1 was ligated to multiple genes participating in carbon catabolism in the GRIL‐seq experiments, among them the carbon catabolism regulator CrcZ, it is possible that a metabolic shift might reduce pyochelin precursors and hence production of the siderophore. The precursor of pyochelin is chorismate, which can also be used for the biosynthesis of pyocyanin, and in the biosynthesis of the aromatic amino acids phenylalanine, tyrosine and tryptophan, or its precursor, anthranilate. Anthranilate can be used for the biosynthesis of alkylquinolones, or directed to the TCA cycle. To investigate these possibilities, RT‐qPCR analysis was conducted on genes of these metabolic pathways, to see if they were affected by overexpressing *lrs1*. Indeed, the mRNA levels of genes for anthranilate synthase *phnAB*, *pqsA*, and the pyocyanin biosynthetic genes were all downregulated in the *lrs1* overexpressing strain (Figures 3B,D,E and S9A,C). The gene *phzS*, which was identified as another potential direct target of Lrs1, was also downregulated, a phenomenon that could be partly through direct interaction with Lrs1. However, genes of anthranilate degradation (*antA*) or tryptophan biosynthesis, *trpEG*, were unaffected (Figures 3B,C and S9A,B). Although anthranilate for alkylquinolone synthesis can be supplied by either the PhnAB or the TrpEG anthranilate synthases, the primary destination of anthranilate produced by TrpEG is tryptophan biosynthesis (Palmer et al. 2013). The carbon catabolism regulatory sRNA CrcZ, another potential target of Lrs1, was upregulated in the *lrs1* overexpressing strain, in line with the hypothesis of Lrs1 involvement in metabolic rewiring (Figure 3F). To corroborate these findings, alkylquinolones were quantified in low iron conditions. Indeed, *lrs1* overexpression significantly reduced the levels of HHQ but did not impact PQS levels, and HQNO was only impacted when the carbon source was succinate (Figure 3G–I). These results indicate that shared metabolites of these pathways are redirected towards either the TCA cycle or aromatic amino acid biosynthesis, deprioritising biosynthesis of pyochelin, alkylquinolones and pyocyanin in low iron conditions.

## 3 Discussion

Post‐transcriptional regulation plays a crucial role in microbial adaptation to environmental changes. A number of sRNAs have already been implicated in controlling the QS systems of *P. aeruginosa* (Oglesby et al. 2008; Sonnleitner et al. 2011; Carloni et al. 2017; Thomason et al. 2019). A previous study has pointed to the sRNA Lrs1 involved in the regulation of the Las QS system in the strain PA14 (Chuang et al. 2019). However, no change in the regulation of *lasR* by Lrs1 was identified in PAO1‐L in the current study. It is noted that this regulation was mainly observed in surface‐attached cells in PA14, whereas here planktonically grown cells were used. Considering that reduced elastase activity was reported in the *lrs1* mutant in PA14 growing under aerobic conditions in LB and reduced *lasR* transcript levels were observed in the same mutant in planktonic cells, it was reasoned that this phenomenon could be observed here too. GRIL‐seq indicated *rsaL* mRNA as a candidate target of Lrs1 although RNA‐seq did not reveal changes in the relative abundance of the *rsaL* mRNA when *lrs1* was overexpressed in PAO1‐L. It is likely that other regulators may mask the effect of Lrs1 in this strain, but *rsaL* may in fact be the missing link in the regulation of *lasR* by Lrs1 in PA14 (Chuang et al. 2019). The strains PAO1 and PA14 are genetically and phenotypically distinct, with regulatory differences between them having been reported before (Grace et al. 2022). This might be the case here too, where Lrs1 has adopted separate roles in the two reference strains, adding to the phenotypic diversity within the species. That would not be the first time that an sRNA does not regulate the same targets in the two strains. The sRNA ErsA was reported to downregulate *algC* in PAO1 only, whereas no regulation could be observed in PA14 (Ferrara et al. 2014). Hence, sRNAs can be drivers of phenotypic and regulatory differentiation between the two strains, and that might expand within the whole *P. aeruginosa* species.

Iron stringency in *P. aeruginosa* is tightly regulated for optimal acquisition and utilisation of this metal. *P. aeruginosa* produces the siderophores pyochelin and pyoverdine for iron uptake, altering between the two depending on the relative abundance of iron in the medium (Dumas et al. 2013). Pyoverdine and pyochelin differ in their affinity for iron, with pyoverdine having a higher Kd than pyochelin, making it more useful in iron scarcity (Brandel et al. 2012). Here, the sRNA Lrs1 may participate in the regulatory switch between the two siderophores in PAO1. Only pyochelin levels were reduced in the lowest iron concentration tested here, with pyoverdine being unaffected in the *lrs1* overexpressing strains. So far, most studies have shown a parallel increase or decrease in the expression of the siderophore biosynthetic operons (Ochsner et al. 2002; Wilderman et al. 2004; Nelson et al. 2019). The only exception has been the sRNA PrrH, which is involved in pyochelin regulation, with the effect being altered in the presence of light or growing the cells statically or aerobically (Hoang et al. 2023). Here, Lrs1 may participate in the differential regulation of the siderophores based on the nutritional needs of the cells. Along with the sRNAs PrrF1, PrrF2 and PrrH, Lrs1 may be involved in the iron stringency response of *P. aeruginosa* by decoupling the synthesis of the two siderophores. By doing so, valuable resources are directed towards essential processes such as pyoverdine and amino acid biosynthesis, increasing the adaptation of the organism and its competitiveness in the environment.

**FIGURE 4:**
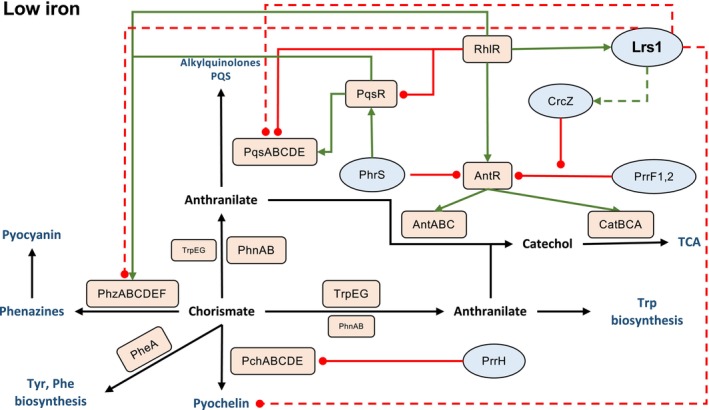
Proposed model of Lrs1 involvement in balancing metabolic flow in low iron in *P. aeruginosa* PAO1‐L. Chorismate is the precursor of the aromatic amino acids, tyrosine, and phenylalanine, as well as pyochelin, pyocyanin, and anthranilate. In turn, anthranilate can be used for the synthesis of the aromatic amino acid tryptophan, the production of alkylquinolones and the QS molecule PQS, or it can be catabolised via the TCA cycle. In low iron, the low‐iron affinity siderophore pyochelin is expressed for iron scavenging, along with increased production of PQS that is used both for QS activation and iron entrapment. The transcription of the PQS biosynthetic genes *pqsABCDE* is activated by the Pqs transcriptional regulator PqsR and indirectly by the PhrS sRNA. At the same time, PhrS downregulates the gene of the anthranilate degradation transcriptional regulator AntR and by extension the anthranilate degradation genes *antABC* and *catBCA* preserving anthranilate for PQS production. The iron stringency response sRNAs PrrF1,2 further repress *antR* sparing precursor molecules for PQS production synthesis while PrrH represses pyochelin biosynthesis. The sRNA CrcZ interferes with the PrrF1,2 mediated repression of *antR*, promoting the degradation of anthranilate for energy conversion. The Rhl transcriptional regulator RhlR further promotes anthranilate degradation by downregulating *pqsR* and *pqsABCDE* while upregulating *antR*. RhlR, along with LasR, upregulate Lrs1 which enhances the effect of RhlR and CrcZ. Lrs1 negatively impacts pyochelin, preserving chorismate for aromatic amino acid biosynthesis. Additionally, Lrs1 downregulates alkylquinolone synthesis through both the *pqs* operon and the anthranilate synthase genes *phnAB* without affecting the anthranilate synthase genes *trpEG* redirecting anthranilate towards either degradation via the TCA cycle or to tryptophan biosynthesis. Finally, Lrs1 increases CrcZ levels, further promoting metabolic efflux to amino acid biosynthesis and energy conversion. Proteins are depicted in orange rectangles and sRNAs in blue ovals. Green lines: positive regulation. Red lines: negative regulation. Red dashed lines: indirect negative regulation. Green dashed lines: indirect positive regulation.

This is also evident by the conditional negative impact of Lrs1 on the Pqs QS system in low iron. PQS can act as an iron scavenger, enhancing the iron uptake response of *P. aeruginosa* (Bredenbruch et al. 2006). Previous studies have indicated that pyochelin and PQS may share the same transporters to enter into the cells (Lin et al. 2017; Zhang et al. 2024). PQS levels remain high under low iron, in part due to the downregulation of the anthranilate degradation transcriptional regulator *antR* by PrrF1,2 and PhrS, providing more precursors for PQS biosynthesis (Djapgne et al. 2018; Dubern et al. 2023; Gebhardt et al. 2023). Lrs1 may provide a regulatory link between pyochelin and PQS, further connecting the PQS QS system to iron uptake. Here, it is proposed that the RhlR‐regulated sRNA Lrs1 amplifies the effects of RhlR and CrcZ on the fates of chorismate and anthranilate (Figure 4). Lrs1 increases the levels of CrcZ possibly through direct interaction, although the mechanism of action has not been fully elucidated. The sRNA CrcZ sequesters Hfq, whereby interfering with the PrrF1,2‐mediated downregulation of *antR* with a possible indirect negative effect on Pqs (Sonnleitner et al. 2017). Since Lrs1 has been shown to interact with Hfq in vitro (Wurtzel et al. 2012), it is likely that the high abundance of this sRNA in the cells may have had secondary effects on the Hfq regulome in vivo as Hfq could be interacting with Lrs1 at the expense of other RNAs.

Additionally, there is the negative effect of RhlR on the Pqs system through repression of *pqsR* and upregulation of *antR* (Xiao et al. 2006b; Oglesby et al. 2008). The pool of available anthranilate for alkylquinolones is reduced as more of it is supplied into the TCA cycle through the actions of the AntABC enzymes. At the same time, the anthranilate synthase genes *phnAB* supplying anthranilate for PQS production are also downregulated. However, the *trpEG* anthranilate synthase genes are unaffected due to their central role in tryptophan biosynthesis (Palmer et al. 2013). Phenazine and pyochelin synthesis is reduced, sparing more chorismate for amino acid biosynthesis. Taken together, Lrs1 may participate in balancing metabolic flow, favouring essential processes such as amino acid biosynthesis and energy conversion in low iron conditions.

In vivo interaction of Lrs1 with multiple mRNAs and sRNAs implicated in diverse metabolic pathways without a clear connection to iron, suggests a more versatile regulatory role for this sRNA. The dysregulation of these pathways was not apparent in the RNA‐seq carried out in the present study, which indicates that regulation may manifest in environmental conditions other than LB. That was the case of CrcZ upregulation, observed here in low iron conditions only. Another hypothesis is that Lrs1 may not act through altering transcript levels of these genes. In many of the targets identified in the GRIL‐seq experiment, Lrs1 was bound to their 3′ UTR, suggesting a distinct function. In a recent study, it was observed that sRNAs were bound to the 3′ UTR of mRNAs in *P. aeruginosa*, among them the 3′ UTR of *oprB* (Gebhardt et al. 2023). It was hypothesised that an sRNA may be transcribed from the 3′ UTR consisting the actual sRNA target, similarly to the 3′ UTR derived sRNA sKatA interacting with PrrF1 and PrrF2 (Han et al. 2016). Here, Lrs1 was also bound to the 3′ UTR of *oprB*, strengthening the possibility of the existence of this sRNA. Collectively, these interactions may indicate a complex regulatory network where sRNAs interact with their targets but also existing in an equilibrium with the other sRNAs and 3′ UTR‐derived sRNAs. The 3′ UTR‐derived sRNA‐sRNA base‐pairing could function as a titrating mechanism to prevent the sRNAs acting upon their targets. That offers a way to controlling the concentration of available sRNA to base‐pair with its targets in the presence of competing sRNAs.

This study highlights the importance of sRNAs for survival of *P. aeruginosa* under nutrient starvation. Here, metabolic fate in low iron seems to be determined by five sRNAs: Lrs1, CrcZ, PhrS and PrrF1,2 acting in opposite ways to each other. The critical role of sRNAs in low iron may be a way for *P. aeruginosa* to navigate an ever‐fluctuating environment where nutrient availability can be stochastic. The sRNAs quickly alter the RNA landscape, favouring the more beneficial metabolic pathways for the current conditions, tipping the balance in favour of *P. aeruginosa* on interspecies competition and infection establishment.

## 4 Experimental Procedures

### 4.1 Strains, Plasmids, Primers, and Culture Media

The bacterial strains and plasmids used in this study are listed in Tables S5 and S6. *P. aeruginosa* strain PAO1‐L was used throughout this study unless otherwise stated. *E. coli* DH5α was used for routine cloning and *E. coli* S17‐1 for conjugal transfers. All strains were grown overnight in lysogeny broth LB at 37°C with shaking at 200 rpm, or on LB agar plates. LB was prepared according to (Sambrook and Russell 2001). For iron‐free conditions, M9 basal salts (Basal salt solution 2× concentrate: 95.9 mM Na_2_HPO_4_, 44 mM KH_2_PO_4_, 34.2 mM NaCl; Supplements 100× concentrate: 1.9 M NH_4_Cl, 20 mM CaCl_2_, 200 mM MgSO_4_) were deferrated with Chelex 100 (50–100 Mesh, Sigma–Aldrich), and the supplements were prepared in iron‐free water. As a carbon source, iron‐free sodium succinate or glucose was added to a final concentration of 20 mM. For low iron conditions, 0.15 μΜ FeCl_3_ was added to the medium, and for iron‐replete conditions, the final ferric iron concentration was 10 μΜ. Antibiotics were added to the media at the following concentrations when necessary: for *E. coli* , 10 μg/mL tetracycline, 10 μg/mL gentamicin, 100 μg/mL ampicillin; for *P. aeruginosa* , 125 μg/mL Tc, 20 μg/mL Gm, 200 μg/mL carbenicillin. Isopropyl β‐D‐1‐thiogalactopyranoside (IPTG) to a 1 mM final concentration was added when required at the start of the culture. Blue‐white selection of colonies was done by adding 5‐bromo‐4‐chloro‐3‐indolyl‐β‐D‐galactopyranoside at 40 μΜ final concentration in agar plates. For the overexpression of *lrs1* from the pKH6 vector, L‐arabinose was added in the medium to a final concentration of 0.2% V/V. Oligonucleotides used in this study are listed in Table S7.

### 4.2 Plasmid Construction

The plasmid pBS_1 was constructed by PCR amplification of a 972 bp region with primers LRS1RV and LRS1FW, which included 615 bp upstream of *pqsA* and 357 bp downstream of the *pqsA* translational start site, and after digestion, it was ligated between the PstI and EcoRI sites of pBluescript.

The plasmid pDP013 was constructed by PCR amplification of the upstream and downstream region of the *lrs1* gene with primer pairs lrs1190delF1FW and lrs1148delF1RV, lrs1190delF2RV and lrs1148delF2FW, respectively. The PCR products were then cloned into PstI‐digested pTS1 with the HiFi DNA assembly kit (NEB). Only the first 138 bp of the *lrs1* gene were deleted, excluding the TSS2, in order to avoid deleting the RpoN binding site situated directly downstream that has been shown to control *pqsA* expression (Shao et al. 2018).

The plasmid pKH6‐lrs1 was constructed by cloning *lrs1* in the vector pKH6. The vector pKH6 contains the arabinose‐inducible P_*bad*_ promoter and has been designed for overexpression of sRNAs (Han et al. 2016). The transcriptional start site of P_*bad*_ is an adenosine and is located 2 bp downstream of an XbaI restriction site (Han et al. 2016). The sRNA gene was amplified with the primers LRS1XBAIFW and LRS1X_ECORI_RV and was subsequently ligated between the XbaI and EcoRI sites of pKH6. An adenosine was added directly upstream of their transcriptional start site in order to allow for correct transcriptional initiation from the P_*bad*_ promoter. The length of cloned *lrs1* was 248 bp as this was previously used in (Chuang et al. 2019).

For the transcriptional promoter fusions in pminiCTXlux, promoter regions were cloned between the HindIII and PstI sites in the MCS of pminiCTXlux. P_*lrs1*_ was amplified with primers LRS1PROHINDIIIF and LRS1PROPSTIRV, P_*lasR*_ with PlasRHindIIIFW and PlasRPSTIRV, P_*lasI*_ with PlasIHindIIIFW and PlasIPSTIRV, and P_*rhlI*_ with PrhlIHindIIIFW and PrhlIPSTIRV. All promoters were amplified from the PAO1‐L gDNA. P_*pqsA*_ was amplified from pBS_1. In the case of translational promoter fusions to pminiCTXlux, DNA fragments containing the promoters were cloned in the XcmI restriction site directly upstream from the translational start site of *luxC* on pminiCTXlux by HiFi DNA assembly (NEB). The promoter P_*pqsA*_ was amplified using the primers PpqsA‐translational‐F and PpqsA‐translational‐R from plasmids pBS_1. The promoter P_*bfrB*_ was amplified with Pbfrb_433_transl_FW and PbfrB_transl_RV.

The pBx‐rne1 plasmid was constructed with Golden Gate assembly. Briefly, the single stranded oligonucleotides sgRNA_1_RV and sgRNA_1_FW were dimerised by incubating equimolar concentrations of each oligonucleotide in annealing buffer (10 mM Tris, pH 8.0, 50 mM NaCl, 1 mM EDTA). The assay was heated to 95°C for 5 min and then the temperature slowly decreased to 25°C. The double stranded oligonucleotide was then incubated with the vector pBx‐Spas‐sgRNA‐Gm and BbsI restriction digestion enzyme in rCutsmart buffer (NEB) for 10 min at 37°C. Afterwards, T4 DNA ligase and 10 mM ATP were added to the assay. The reaction was incubated for 20 cycles of alternate steps, 3 min at 37°C and 3 min at 16°C.

The plasmid pME6032‐*rhlR* was constructed by cloning the open reading frame of *rhlR* between the EcoRI and KpnI sites, with transcription and translational initiation provided by the P_*tac*_ promoter in pME6032. The gene *rhlR* was amplified with RhlR‐C‐F and RhlR‐C‐R from the genome of PAO1‐L.

The suicide plasmids pME3087‐rhlR, pME3087‐rhlI, pME3087‐lasR, pME3087‐lasI and pME3087‐pqsE for the construction of deletion mutants of the genes *rhlR, rhlI, lasR*, and *lasI, pqsE* respectively, were constructed as follows. The flanking regions of each gene were amplified with their respective primers and were individually cloned in pBluescript, at which point the constructs were verified by sequencing. Then, the upstream and downstream regions were cloned and ligated together in pBluescript at their mutual XbaI site, or BamHI for the pME3087‐pqsE, with restriction digestion and ligation. The resulting fragment was transferred to the suicide vector pME3087 that was then used for the construction of the respective deletion mutants.

### 4.3 Strain Construction

All plasmids were transferred to *E. coli* electrocompetent cells prepared according to Sambrook and Russell (2001). *P. aeruginosa* electrocompetent cells were prepared according to Choi et al. (2006).

For deletion mutants, the corresponding suicide plasmids were transformed in *E. coli* S17‐1 and subsequently conjugated to PAO1‐L. Following conjugation, strains carrying a pTS1‐ or pEX18‐based suicide vector were sucrose counter‐selected for double recombinants according to the protocol from Huang and Wilks (2017). Colonies resistant to sucrose and susceptible to the corresponding antibiotic were PCR screened to identify the mutants, followed by sequencing to confirm the deletion. For the construction of deletion mutants with pME3087‐based suicide vectors, the tetracycline‐sensitive enrichment approach was followed. Briefly, cells from an overnight culture were incubated in LB for 2 h; 10 μg/mL tetracycline was added for 1 h, followed by the addition of 300 μg/mL carbenicillin for 6 h. This process was repeated three times, after which tetracycline‐sensitive colonies were selected and screened by PCR. The pminiCTXlux suicide plasmids carrying the promoter fusions were conjugated to *P. aeruginosa* from *E. coli* S17‐1.

The plasmid pUC18‐miniTN7‐Plac‐dCas9‐Gm was transferred to PAO1‐L by triparental conjugation with the helper vector pTNS1. Following insertion of the Tn7 construct into the chromosome, the gentamicin resistance cassette was excised by electroporating the flipase‐encoding plasmid pFLP2. Curation of pFLP2 was done by sucrose counterselection as described in Huang and Wilks (2017). Afterwards, pBx‐Spas‐sgRNA‐Gm and pBx‐rne1 were electroporated to the gentamicin‐sensitive strain.

### 4.4 Bioluminescence Assays

Promoter activity was determined using pminiCTXlux bioluminescent reporters integrated to the *P. aeruginosa* chromosome. Cultures were grown overnight in LB with appropriate antibiotics. The overnight cultures were diluted to a final OD_600_ = 0.01 and 200 μL were added in each well in a 96‐well plate (flat black, transparent bottom, GreinerBioOne), in triplicates, and the plate was covered with a transparent lid. Measurements of OD_600_ and relative luminescence units RLU were taken every 30 min for 24 h at 37°C unless otherwise stated in the figure legend. Promoter activity was calculated as RLU/OD_600_. Statistical analysis was performed with the software Graphpad Prism.

### 4.5 Pyoverdine and Pyochelin Quantification

In 100 mL flasks, 10 mL iron‐free M9 minimal media with 20 mM glucose was inoculated with 0.05%V/V overnight cultures and incubated for 20 h at 37°C, 200 rpm. Following centrifugation to pellet the cells, the supernatants were filter‐sterilised. Pyoverdine was quantified by measuring the absorbance of the supernatant at 400 nm. Pyochelin was quantified according to Cunrath et al. (2020). Briefly, 1 mL of supernatant was acidified with 50 μL 1 M citric acid. Then, pyochelin was extracted twice with 500 μL ethyl acetate and the absorbance was measured in a quartz cuvette at 320 nm. Both OD_400_ and OD_320_ were divided by OD_600_ to account for cell culture density.

### 4.6 Elastase Quantification

In 50 mL flasks, 10 mL LB was inoculated with 0.05% V/V overnight cultures and was incubated for 20 h at 37°C, 200 rpm. 1.5 mL of culture was centrifuged to pellet the cells, and the supernatant was filter sterilised. 100 μL of the supernatant was mixed with 900 μL ECR buffer (1.41 g/100 mL Tris, 19.47 mg/100 mL CaCl_2_, pH 7.5) with 30 mg/mL elastin congo red in eppendorfs. The mix was incubated at 37°C, 200 rpm for 4 h. Then, the eppendorfs were centrifuged at 13,000 g for 3 min, and the absorbance of the supernatant was measured at 495 nm.

### 4.7 Quantification of AQs and Anthranilate

The comparative analysis of alkyl quinolones in supernatant samples was conducted by liquid chromatography—tandem mass spectrometry (LC–MS/MS) using an Excion LC system in tandem with a Sciex Qtrap 6500+ mass spectrometer. Analytical samples were prepared by mixing 10 μL of sterile filtered supernatants with 90 μL of an internal standard solution (500 nM solution of deuterated HHQ (d4‐HHQ) in MeOH). 5 μL of each sample was injected onto a Phenomenex Gemini C18 column (3.0 μm, 50 × 3.0 mm^2^), and eluted at an LC flow rate of 0.45 mL/min. A linear binary gradient (mobile phase A: 2 mM 2‐picolinic acid in 0.1% (V/V) formic acid, mobile phase B: 0.1% (V/V) formic acid in MeOH) from 10% B to 99% B over 5 min, followed by a further 2 min at this composition was used. AQ detection was conducted with the MS operating in MRM (multiple reaction monitoring) mode, screening the LC eluent for the specific analytes of interest.

### 4.8 Northern Blots

Total RNA was extracted from cell cultures using the RNeasy plus Universal mini kit (Qiagen) following the manufacturer's instructions. For the detection of Lrs1 by Northern Blot, the DIG (digoxygenin) Northern Starter kit (Roche) was used following the manufacturer's instructions with some modifications. The endogenous control was 5S rRNA. The probes for both Lrs1 and 5S were created with in vitro transcription of PCR templates for both genes. For Lrs1 detection, 10 μg RNA was loaded per sample and 200 ng for 5S rRNA. The samples were loaded in 10.5% V/V vertical denaturing polyacrylamide gels with 5 M urea and 1× TBE. The samples were mixed with an equal volume of denaturing RNA loading buffer. As a marker, the Century‐RNA marker templates (Invitrogen) were in vitro transcribed and incorporated DIG‐11‐UTP. The gels were electroblotted on positively charged nylon membranes (Amersham Hybond‐N RPN 203 N) followed by UV crosslinking and hybridised in DIG EasyHyb buffer (Roche) at 68°C with gentle agitation overnight. Following incubation with anti‐DIG antibody, the membranes were incubated with the substrate CDP‐star ready‐to‐use solution (Roche). The membranes were finally developed by exposing them to X‐ray film.

### 4.9 Quantitative Real Time PCR (RT‐qPCR)

Following RNA extraction, the samples were treated with Turbo DNA‐free kit to remove residual DNA. The RNA was reverse transcribed with the GoScript Reverse Transcriptase kit with random primers (Promega). The qPCR mix used here was the Power SYBR Green PCR Master Mix (Applied Biosystems, Cat: 4367659). The qPCRs were performed in a QiaQuant 5× multiplex system. The *rpoD* mRNA was chosen as an endogenous control. Three biological replicates and three technical replicates were analysed for each target. ΔΔCt ratios were calculated with the equation described by Pfaffl (2001) and data were analysed on GraphPad Prism.

### 4.10 Electrophoretic Mobility Shift Assay (EMSA)

The RNAs were transcribed in vitro with the MegaScript T7 Transcription kit (Invitrogen) from PCR templates. The templates were made for each RNA with a forward primer containing the T7 promoter in the 5′ end. Subsequently, the RNAs were purified with the RNeasy cleanup kit (Qiagen). A non‐radioactive approach was followed for the visualisation of EMSA. The tagged RNA and Lrs1 was made with a reverse primer starting with the sequence 5′ TTTTTTTTCCCCCCCCC 3′. This sequence would add a 5′ GGGGGGGGGAAAAAAAA 3′ which was complementary to a probe (the Atto probe) covalently bound to the fluorophore Atto_700_. Atto_700_ fluoresces at the infrared, with peak excitation at 699 nm and peak emission at 716 nm. The sRNA and the Atto‐probe were mixed at a 1:5 molar ratio annealing buffer (10 mM Tris–HCl pH = 7.5, 50 mM NaCl, 1 mM EDTA). The reaction was boiled for 5 min at 95°C and it was gradually cooled to 25°C. The RNA targets were diluted in water to a concentration of 1 μΜ and boiled for 1 min at 95°C, chilled on ice for 1 min, and transferred at room temperature for 5 min to allow for RNA folding (Bak et al. 2015). The probed sRNA and the target RNA were mixed together in dimerization buffer (10×: 200 mM Tris–HCl pH = 7.5, 100 mM NaCl, 250 mM MgCl_2_, 10 μΜ yeast tRNA) in 10 μL reactions. The probed sRNA concentration was constant at 10 nM, and the target RNA was at 0, 10, 25, 50 and 100 nM. The reactions were incubated for 30 min at 37°C and the reaction was stopped by addition of 2 μL non‐denaturing loading buffer (12% glycerol, 1 × TBE, 0.001% w/V bromophenol blue). The whole reaction was loaded in 5% non‐denaturing PAGE. The gels were run in 0.5 × TBE, at 50 V, 2 h, at 4°C, in the dark. The gels were visualised at the Li‐cor Odyssey Infrared Imaging System.

### 4.11 RNA Sequencing

RNA samples for RNA‐sequencing were harvested as follows. In 50 mL flasks, 10 mL LB with 0.2% L‐arabinose was inoculated with 0.05% V/V overnight culture. The cultures were incubated at 37°C, 200 rpm. Exponential phase samples were taken when cultures reached an OD_600_ ~ 0.5, and early stationary phase when they reached OD_600_ ~ 3.3.

The RNA sequencing was conducted in the DeepSeq Facilities at the University of Nottingham. The library pool was sequenced on the Illumina NextSeq 500 using a NextSeq 500 high Output 150 cycle kit (Illumina) to generate over 10 million pairs of 75 bp paired‐end reads per sample.

### 4.12 GRIL‐Seq

The sample collection and preparation for RNA‐Seq was performed as described in the analytical protocol in the supporting files of Han et al. (2016). In 250 mL flasks, 30 mL LB containing Cb_400_ and Gm_20_ was inoculated with overnight cultures to a final OD_600_ = 0.01. The flasks were incubated at 37°C, 200 rpm until OD_600_ ~ 0.5 (exponential phase samples) at which point the cultures were split. 10 mL were added in two 50 mL flasks and 10 μL IPTG 1 M (C_final_ = 1 mM) was added in one flask (test flask, induced conditions) to induce expression of T4 RNA ligase and nothing was added in the other (control flask, uninduced conditions). An hour later, L‐arabinose was added in the test flask to induce the sRNA transcription from the pKH6 vector. 20 min later, 1.6 mL samples were collected. The same procedure was followed for the stationary phase samples, with the induction starting when OD_600_ ~ 3.3. Following sRNA enrichment (Han et al. 2016), the RNA was sequenced at the DeepSeq facilities at the University of Nottingham as described for the RNA‐sequencing method above with some modifications. The library pool was sequenced on the Illumina NextSeq500 over one NextSeq500 Mid Output 300 cycle kit (Illumina), to generate approximately 20 million pairs of 150‐bp paired‐end reads per sample.

Data analysis was conducted on the online server Galaxy (usegalaxy.eu). The reads were locally aligned to the corresponding sRNA sequence only using Bowtie2 (v2.5.0, galaxy version 2.4.5 + galaxy1) (Langmead et al. 2009) with the following parameters changed: allowed mismatches in the multiseed alignment: 1, length of the seed substrings: 20, match bonus: 3. The aligned reads were subjected to a further local alignment with Bowtie2, preserving the same parameters. This time, the genome for the alignment was that of PAO1, with the sequences of the sRNA and the rRNAs deleted. Mapped reads were counted with FeatureCounts (galaxy version 2.0.3 + galaxy1) (Liao et al. 2014). The mapped alignments were visualised with Artemis (https://www.sanger.ac.uk/tool/artemis/). The prediction of sRNA–RNA seed regions was conducted with the online tool IntaRNA 2.0 (http://rna.informatik.uni‐freiburg.de/IntaRNA/Input.jsp) (Busch et al. 2008; Mann et al. 2017). The alignment of the chimeric reads to a manually made chimeric sequence of the sRNA and the mRNA was done on Benchling (https://benchling.com/) using the MAFFT aligner (Katoh and Standley 2013) with the default parameters.

### 4.13 Genome Sequencing

The WT PAO1‐L and the *lrs1* mutant Δlrs1 were whole‐genome sequenced at MicrobesNG (https://microbesng.com). SNP and small indels were called by the pipeline of MicrobesNG, and genome assemblies were visualised on IGV (Robinson et al. 2011). The accession number of the WT PAO1‐L was NZ_CP099798.

## Author Contributions

**Dimitra Panagiotopoulou:** writing – original draft, investigation, methodology, validation, visualization, formal analysis, data curation, conceptualization, funding acquisition. **Natalia Romo Catalán:** conceptualization, investigation, funding acquisition. **Max Wilcox:** investigation. **Nigel Halliday:** investigation, methodology. **Paolo Pantalone:** investigation. **James Lazenby:** investigation. **Miguel Cámara:** conceptualization, writing – review and editing, supervision, resources, funding acquisition. **Stephan Heeb:** conceptualization, writing – review and editing, supervision, resources.

## Conflicts of Interest

The authors declare no conflicts of interest.

## Supporting information


**Data S1.** Supporting Information.

## Data Availability

All transcriptomic data used in this study have been submitted to the European Nucleotide Archive (ENA) under the accession numbers: PRJEB82486 for the RNA‐seq data and PRJEB82615 for the GRIL‐seq data.
